# War on Pallagra

**Published:** 1912-09

**Authors:** 


War on Pallagra.—A representative from Tennessee has, in the last few days, introduced a bill in Congress calling for the instruction of experts of the United States Public Health Service to investigate the causes and discover the cure for pellagra. If passed, this bill would carry an appropriation of $50,000 for this purpose.—*Va. Semi-Monthly.*

---

Dr. William F. Drewry, of Petersburg, Va., was recently elected a member of the National Committee of the International Congress of Medicine, which is to be held in London, England, August 6-12, 1913. This committee is composed of representative men in the various branches of medicine from different parts of the United States.—*Va. Semi-Monthly.*

